# Complex nonlinear capacitance in outer hair cell macro-patches: effects of membrane tension

**DOI:** 10.1038/s41598-020-63201-6

**Published:** 2020-04-10

**Authors:** Joseph Santos-Sacchi, Winston Tan

**Affiliations:** 1 Surgery (Otolaryngology), Yale University School of Medicine, 333 Cedar Street, New Haven, CT 06510 USA; 2 Neuroscience, Yale University School of Medicine, 333 Cedar Street, New Haven, CT 06510 USA; 3 Cellular and Molecular Physiology, Yale University School of Medicine, 333 Cedar Street, New Haven, CT 06510 USA

**Keywords:** Biophysics, Membrane biophysics

## Abstract

Outer hair cell (OHC) nonlinear capacitance (NLC) represents voltage sensor charge movements of prestin (SLC26a5), the protein responsible for OHC electromotility. Previous measures of NLC frequency response have employed methods which did not assess the influence of dielectric loss (sensor charge movements out of phase with voltage) that may occur, and such loss conceivably may influence prestin’s frequency dependent activity. Here we evaluate prestin’s complex capacitance out to 30 kHz and find that prestin’s frequency response determined using this approach coincides with all previous estimates. We also show that membrane tension has no effect on prestin’s frequency response, despite substantial shifts in its voltage operating range, indicating that prestin transition rate alterations do not account for the shifts. The magnitude roll-off of prestin activity across frequency surpasses the reductions of NLC caused by salicylate treatments that are known to abolish cochlear amplification. Such roll-off likely limits the effectiveness of prestin in contributing to cochlear amplification at the very high acoustic frequencies processed by some mammals.

## Introduction

Prestin (SLC26a5) underlies outer hair cell (OHC) mechanical activity^1^, whereby voltage-dependent conformational transitions couple into length changes of the cell (electromotility; eM)^2–4^. Sensor charge movements associated with these conformational changes are measurable as an electrical correlate of eM, i.e., nonlinear capacitance (NLC)^5–7^, which is maximal at V_h_, the voltage where prestin charge is distributed equally across the OHC membrane and where eM gain is greatest.

The study of OHC NLC by admittance techniques in whole cell voltage clamp is compromised by contributions from stray capacitance, membrane conductances, and electrode series resistance (R_s_), the latter altering over time due to plugging of the patch pipette tip with intracellular constituents. Because of this, it is virtually impossible to measure complex membrane capacitance in whole cell mode. We avoid these issues by measuring membrane admittance from macro-patches of the OHC lateral membrane. The lateral membrane of the cylindrical OHC is dominantly populated by prestin (>8000 functional units/μm^2,8–10^), whereas voltage-dependent membrane conductances are housed in the basal pole membrane^11^. Consequently, macro-patch admittance, following removal of stray capacitance by subtraction of admittance at very depolarized levels where NLC is absent^12^, can be used to study complex sensor-charge movements arising from voltage-induced conformational changes in prestin.

Here, we evaluate patch admittance to provide estimates of complex capacitance representing charge movements both in phase and 90° out of phase with AC voltage excitation. We compare such data to previously obtained measures of OHC NLC frequency response^13,14^, and additionally report on the effects of membrane tension^15–17^ on the frequency response of complex NLC. Will an assessment of NLC based on measures of complex NLC alter our current view, as suggested^18^, and can turgor pressure, normally present in the OHC^19^, with its effect on membrane tension be influential? Our observations indicate that the bulk of sensor charge movement is in phase with voltage, while the resistive component (dielectric loss) is relatively small. Furthermore, membrane tension, though altering prestin’s operating voltage point, has no effect on its frequency response. Thus, magnitude estimates of complex NLC are comparable to those measured with other methods^13,14^, being unusually low pass in nature (non-Lorentzian) and indicating that the absolute movement of prestin charge that drives electromotility (eM)^20,21^ is unlikely to extend with high fidelity to the very high acoustic frequencies (60–160 kHz) detected by some mammals.

## Methods

All experimental protocols were approved by the Yale Animal Care and Use Committee, and were in accordance with relevant guidelines and regulations. Methods, including details of our voltage chirp stimulus protocol, are detailed in^13^. Briefly, extracellular solution was (in mM): NaCl 100, TEA-Cl 20, CsCl 20, CoCl_2_ 2, MgCl_2_ 1, CaCl_2_ 1, Hepes 10, pH 7.2. Experiments were performed at room temperature. Extracellular solution was in the patch pipette. On-cell macro-patches on the guinea pig OHC lateral membrane were made near the middle of the cylindrical cell, since prestin density/activity is uniform within the lateral membrane^8,22^. We have previously shown that capacitance frequency response in the OHC patch remains the same after excision of the patch, indicating that on-cell recording is applicable for our purposes^13^. Borosilicate pipettes of inner tip dimeters between 3–4 μm were used, with M-coat applied within about 20 μm of the tip to minimize pipette capacitance. In order to establish gigohm seals we supplemented extracellular solution with 5μM Gd^3+^; we have shown previously that theses low concentrations help to form seals without affecting NLC^13,23^. In 2 on-cell patches, we have omitted Gd^3+^ in the pipette solution and NLC frequency response is not significantly different than in its presence. An Axon 200B amplifier was used with jClamp software (www.scisoftco.com). An Axon Digidata 1440 was used for digitizing at 10 μs (Nyquist frequency of 50 kHz), with a 4-pole Bessel filter of 10 kHz. Membrane admittance was determined using a series of voltage chirps (4096 points each, resolution 24.4 Hz) superimposed onto holding potentials ranging from −160 to 160 mV, in 40 mV increments. 100 ms after each step to a new holding potential, 26 contiguous chirp-induced current responses at each holding potential were time-averaged. Real and imaginary components of the membrane admittance at all chirp frequencies were determined by FFT in jClamp, and corrected for the roll-off of recording system admittance^13,24^. This correction controls for all attenuation and phase characteristics of the system, including the filter, as we have shown previously^13^, providing valid estimates of capacitance beyond the filter cut-off. Our dual-sine methodology permitted us to measure capacitance with high fidelity out to 20 kHz across holding potentials^13^. Here using single sine analysis we extend that to 30 kHz.

Our patch pipette inner tip diameters (see Results) were used to estimate the linear capacitance of membrane patches. We estimate a linear membrane patch capacitance of 187.8 +/− 15.4 fF (n = 25). This was determined by estimating membrane patch hemispheric surface area using the standard value of 1 μF/cm^2^ ^25^. In our presentation below, we provide absolute estimates of NLC, and specific estimates of NLC by dividing patch admittance with linear capacitance for each patch, thereby accounting for different patch size. Patch experiment data were accepted for inclusion if maximum NLC within our recording bandwidth was >150 fF. Complex values were first averaged for all average-based analyses. Plot traces are smoothed with a 6 point (150 Hz bandwidth) running average in Matlab.

In order to extract Boltzmann parameters, capacitance-voltage data were fit to the first derivative of a two-state Boltzmann function.

m1 $${C}_{m}=NLC\,+{C}_{sa}+{C}_{{\rm{lin}}}={Q}_{{\rm{\max }}}\frac{{\rm{ze}}}{{k}_{B}T}\frac{b}{{(1+b)}^{2}}+{C}_{sa}+{C}_{{\rm{lin}}}$$

where

$$b=exp\left(-ze\frac{{V}_{m}-{V}_{h}}{{k}_{B}T}\right),\,{C}_{sa}=\,\frac{\Delta {C}_{sa}}{(1+{b}^{-1})}$$

Q_max_ is the maximum nonlinear charge moved, V_h_ is voltage at peak capacitance or equivalently, at half-maximum charge transfer, V_m_ is R_s_-corrected membrane potential, *z* is valence, C_lin_ is linear membrane capacitance, e is electron charge, *k*_*B*_ is Boltzmann’s constant, and T is absolute temperature. C_sa_ is a component of capacitance that characterizes sigmoidal changes in specific membrane capacitance^12,26^. Functional prestin density in the membrane is based on quantity of sensor charge.

m2 $${Q}_{max}=\left[\frac{4\cdot {k}_{B}\cdot T}{z\cdot e\,}\right]\cdot \,NL{C}_{Vh}$$

m3 $$Prestin\,density=\frac{{Q}_{max}}{patch\,surface\,area}$$

A power fit of NLC across frequency (*f*) was performed^13,27^.

m4 $$NLC(f)=NL{C}_{{0}}+a\ast {f}^{b}$$

where NLC_0_ is the zero frequency component, and *a* and *b* control the frequency response.

## Results

In our present study, we successfully recorded from 25 macro-patches on the OHC lateral membrane, where membrane breakdown did not occur; breakdown might be expected beyond our voltage protocol voltages^28^. Our pipette inner tip diameter was 3.45 +/− 0.15 μm. R_s_ of pipettes (under slight positive pressure) in bath prior to patching, determined by step voltages, was 1.34 +/− 0.04 MΩ. However, this may not reflect series resistance following patch configuration. We therefore calculated R_s_ given a typical resistivity for pipette solutions of 100 ohm-cm^29^, and our pipette taper angle of 0.2 rad; we calculate an R_s_ of about 920 kΩ. Since the patch membrane extends into the pipette where pipette diameter is larger, we further estimate a reduction of R_s_ to 735 kΩ. The difference in NLC frequency response provided between measured and calculated R_s_ were minimal, showing a −2.2 dB reduction in NLC at 30 kHz for the directly measured values. Below we utilize the calculated value response.

The lateral membrane of the OHC is virtually devoid of voltage-dependent conductances^11^, and we additionally use channel blockers to insure this. Our seal resistance, determined by linear fit of step induced currents within a linear region of the I-V between −40 and +40 mV, was 5.39 +/− 0.65 GΩ.

Consequently, the macro-patch membrane, unlike the membrane in whole cell conditions, may be considered an isolated capacitor under voltage clamp, and thus amenable to determination of complex capacitance. In the following analysis of complex capacitance, we follow the methodology of Fernandez *et al*.^30^, applied to each patch individually, based on its characteristics. The OHC patch capacitance presents as a parallel combination of linear (C_lin_) and prestin-generated (NLC) capacitance. Conceivably, prestin’s voltage-sensor may work as an imperfect, lossy capacitor that possesses both resistive (due to dielectric loss) and capacitive components (e.g., modelled as a combination of capacitor and resistor); that imperfection may influence estimates of NLC^18^. In addition to biophysical capacitance, system-generated stray capacitance (C_stray_) will contribute to our measures. C_stray_, though voltage-independent, may also possess resistive and capacitive components, its admittance being $\,{Y}_{stray}^{\ast }\,(\omega )$. Under voltage clamp, an AC voltage across the patch membrane (V_m_) induces an AC current (I_m_), where the admittance (*Y*_*m*_ = I_m_/V_m_) is a complex function of angular frequency, $\,\omega =2\pi f\,{\rm{and}}\,i=\sqrt{-1\,}$

1 $${Y}_{m}(\omega )={G}_{m}(\omega )+i{B}_{m}(\omega )$$

with *G*_*m*_ representing membrane conductance, *B*_*m*_ representing membrane susceptance. Before continuing, we remove the effects of series resistance (*R*_*s*_) by subtracting it from the real component of membrane impedance ${Z}_{m}(\omega )(1/{Y}_{m}(\omega ))$, and then converting back to admittance^30^. ${Y}_{m}(\omega )$ can be described in more detail,

2 $${Y}_{m}(\omega )={G}_{m}(\omega )+\,{G}_{leak}\,+\,{Y}_{stray}^{\ast }\,(\omega )+\,i\omega \,{C}_{m}(\omega )$$

where C_m_ (*ω*) = C_lin_ + NLC.

*G*_*leak*_ represents a DC leakage conductance. *C*_*lin*_ is taken as frequency independent and via small signal analysis we seek to determine the frequency dependence of NLC, after removing $\,{Y}_{stray}^{\ast }\,(\omega )$. To our benefit, admittance at +160 mV lacks NLC^12^

3 $$\,{Y}_{m}^{160}(\omega )={G}_{m}(\omega )+\,{G}_{leak}+{Y}_{stray}^{\ast }\,(\omega )+\,i\omega \,{C}_{lin}$$

Thus, subtraction of membrane admittance at +160 mV from those corresponding measures at all other holding potentials provides a differential admittance, ${Y}_{m}^{\ast }(\omega )$, devoid of stray capacitance effects.

4 $${Y}_{m}^{\ast }(\omega )=\,{Y}_{m}(\omega )-{Y}_{m}^{160}(\omega )={G}_{m}^{\ast }(\omega )+i{B}_{m}^{\ast }(\omega )$$

Actually, after subtraction, there remains a small differential residual nonlinear, voltage-dependent DC leakage conductance (*G*_*leak*_), which we remove from ${G}_{m}^{\ast }(\omega )$ by subtraction of the real part of ${Y}_{m}^{\ast }(\omega )$ at zero frequency. We determine this value to subtract by extrapolating to zero frequency with a linear fit of ${\rm{Re}}({Y}_{m}^{\ast }(\omega ))$ between 24.41 and 463.86 Hz at each of the stepped holding potentials. This is akin to removing leakage conductance determined by prior DC step estimates, but has the further advantage of being determined during the actual chirp stimulation period.

Complex membrane capacitance, a function of angular frequency, is defined as (see Fernandez *et al*.^30^)

5 $$\begin{array}{c}{C}_{m}^{\ast }(\omega )=\,\frac{{Y}_{m}^{\ast }(\omega )}{i\omega }=\,\frac{{B}_{m}^{\ast }(\omega )}{\omega }\,-i\,\frac{{G}_{m}^{\ast }(\omega )}{\omega }=\,{C}_{m}^{\ast }{\rm{{\prime} }}(\omega )-i\,{C}_{m}^{\ast {\rm{{\prime} }}{\rm{{\prime} }}}(\omega )\end{array}$$

**Figure 1:**
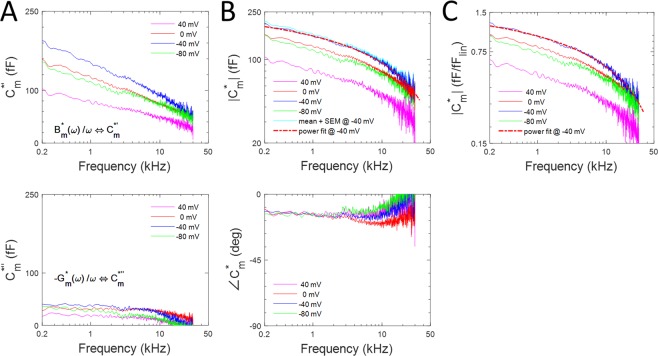
Complex capacitance of OHC lateral membrane macro-patches. (**A)** Top panel shows the mean real components of complex capacitance at selected holding potentials; bottom panel shows corresponding imaginary parts. (**B)** Top panel plots mean magnitudes of complex capacitance at different holding potentials. A power function fit of the magnitude at the −40 mV holding potential is shown by the dotted line (red) overlying the mean (dark blue line). The light blue line is the mean + SEM at that same holding potential data. Fit parameters: NLC_0_: 279.17 fF, a: −34.35, b: 0.18. Bottom panel show the phase of complex capacitance. (**C)** Plot of the means of complex capacitance magnitude per estimated linear patch capacitance (see Methods). Fit parameters: NLC_0_: 1.71 fF, a: −0.19, b: 0.19. Note low-pass behavior of magnitude functions.

In Fig. 1A, we plot the average capacitive (real, ${C}_{m}^{\ast }{\prime} (\omega ))$ and apparent conductance (imaginary, ${C}_{m}^{\ast {\prime\prime} }(\omega ))\,$ components of the complex capacitance for holding potentials of 40, 0, −40 and −80 mV. The values are voltage dependent. While the capacitive component is large and frequency dependent, the conductance component is smaller and less frequency dependent, differing from a larger component predicted from diffusion-based charge translocation^18^.

The absolute magnitude of the complex capacitance can be used to glean an estimate of membrane capacitance $({C}_{m}^{1}(\omega ))$,

6 $${C}_{m}^{1}(\omega )=|{C}_{m}^{\ast }(\omega )|$$

In Fig. 1B, the absolute magnitude, $|{C}_{m}^{\ast }(\omega )|\,$, of the complex capacitance is plotted. The lower panel plots the phase angle. The magnitude rolls off continuously across frequency at each holding potential. In Fig. 1C, the data are plotted as a specific complex capacitance, i.e., per linear capacitance of the patches. As found in whole cell recordings, peak NLC can be larger than linear capacitance^5,6^.

It is also possible to estimate capacitance of the macro-patch membrane by a “phase tracking” approach. That is, the phase angle of the complex admittance ${Y}_{m}^{\ast }(\omega )$ can be rotated to minimize its real component (${G}_{m}^{\ast }(\omega )$), providing a new value ($({Y}_{m}^{\ast \ast })$), whose imaginary component reflects the capacitive component (susceptance) of the admittance in the absence of conductance interference. Another estimate of membrane capacitance, ${C}_{m}^{2}(\omega )$, can then be obtained.

7 $$\angle ({Y}_{m}^{\ast }(\omega ))={\rm{atan}}\left(\frac{{B}_{m}^{\ast }(\omega )}{{G}_{m}^{\ast }(\omega )}\right)$$

8 $${Y}_{m}^{\ast \ast }={Y}_{m}^{\ast }(\omega )\,\cdot \,{e}^{(-i\left(\angle ({Y}_{m}^{\ast }(\omega ))-\frac{\pi }{2}\right)}$$

9 $${C}_{m}^{2}(\omega )=\frac{{\rm{Im}}({Y}_{m}^{\ast \ast })}{\omega }$$

This approach is similar to traditional real time capacitance phase tracking under voltage clamp^31^, where the capacitive component at the angle of δY/δC_m_^32^ is obtained by adjusting the lock-in recording angle until the conductance component is minimized and the capacitive component is maximized. In that approach, calibration with a known capacitance provides membrane capacitance estimates at the measurement frequency. This approach to measure OHC patch NLC was used by Gale and Ashmore^14^.

**Figure 2:**
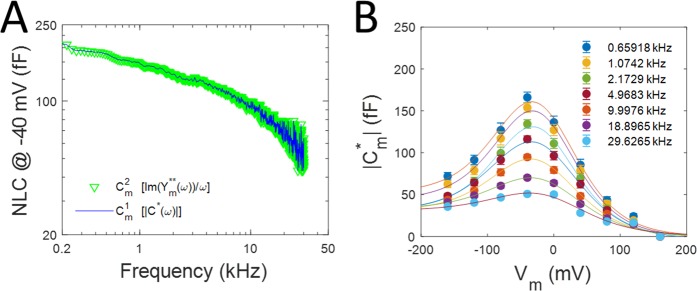
Comparison of two estimates of NLC frequency response at −40 mV holding potential. (**A**) Each estimate provides equivalent results. (**B**) C-V plot of complex capacitance magnitude of OHC lateral membrane macro-patches at selected frequencies (mean +/− SEM). Data are fit (Eq. m1) to extract Boltzmann parameters (see Methods). −40 mV is close to V_h_. From lowest to highest frequency fit values are (peak NLC, V_h_, z): 133.60, 127.61, 115.42, 98.81, 76.81, 52.60, 34.35 fF; −25.2, −25.3, −26.0, −26.4, −26.9, −20.6, −16.4 mV; 0.61, 0.62, 0.62, 0.57, 0.59, 0.56, 0.54. Prior to averaging patch responses (n = 25), capacitance values from each patch were obtained by averaging over a bandwidth of about 200 Hz about the listed frequency value.

In Fig. 2A, we plot the two estimates of OHC NLC $[{C}_{m}^{1}(\omega ),\,and\,{C}_{m}^{2}(\omega )]$, corresponding to methods utilizing the complex capacitance magnitude, and the phase tracking approach, respectively – both at −40 mV holding potential (near V_h_). The measures overlap, indicating that Eqs. 6 and 9 return the same result. In Fig. 2B, the voltage dependence of the complex capacitance magnitude at selected frequencies, presents a bell-shaped function typical of OHC NLC^5,6^, whose peak precipitously decreases with frequency, but whose voltage at peak (V_h_) remains similar across frequency. These data are fit (Eq. m1) to extract the Boltzmann parameters given in the legend. Prestin density based on fits to capacitance at 1 kHz is 1133/μm^2^, similar to previous estimates at a similar frequency^8–10^.

Having arrived at practical approaches to estimate OHC patch NLC, we now look at the influence of membrane tension. Several studies under whole cell voltage clamp have found that as membrane tension is increased, NLC V_h_ shifts in the depolarizing direction, and decreases in peak magnitude^15–17^. However, utilizing lock-in estimates of lateral membrane patch capacitance instead of the whole cell technique, Gale and Ashmore^9^ found no evidence for a reduction in peak capacitance despite shifts in the depolarizing direction.

**Figure 3:**
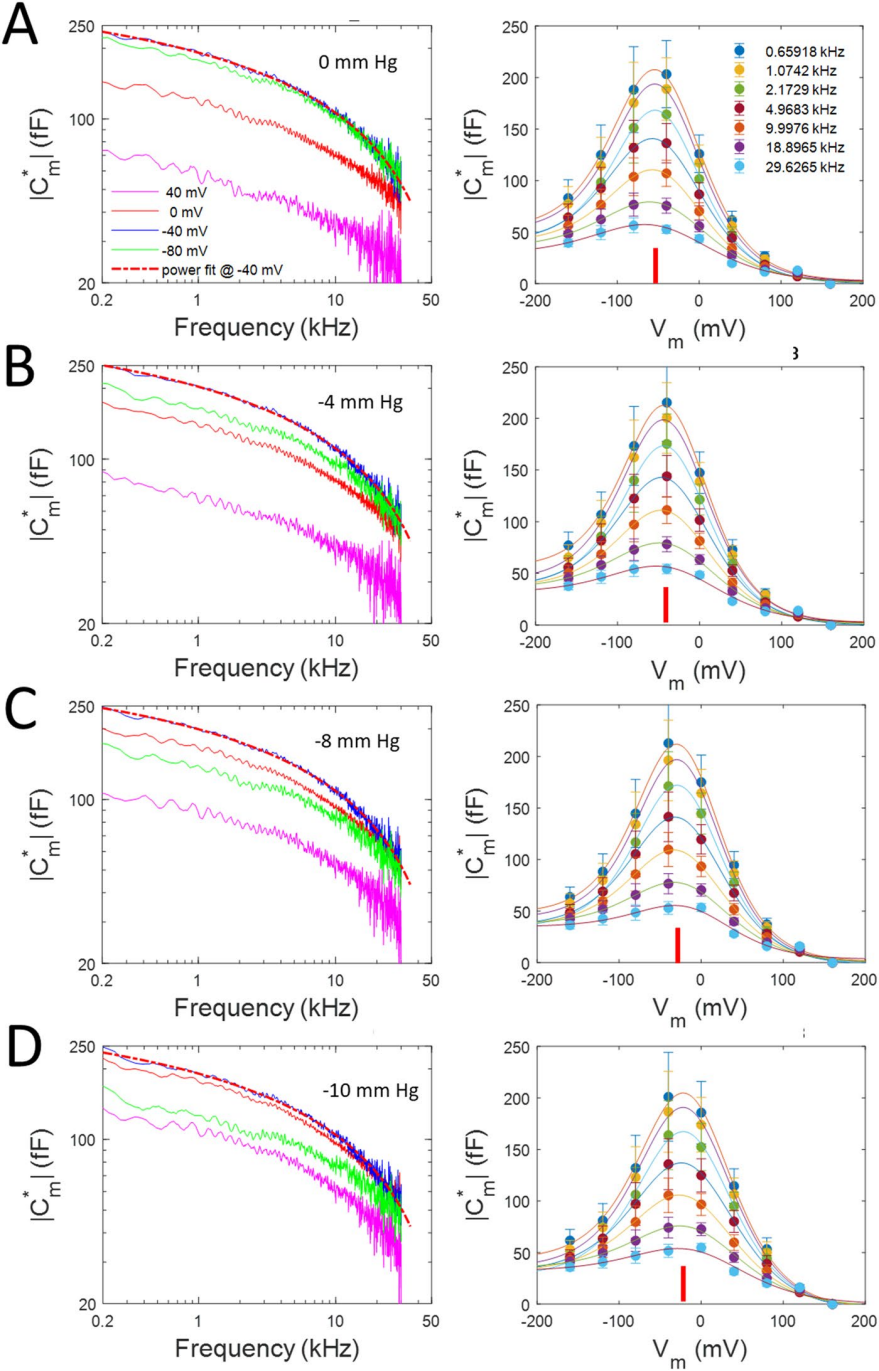
Effect of membrane tension on magnitude of complex capacitance voltage and frequency dependence. Panels (A–D) depict results at 0, −4, −8 and −10 mm Hg pipette pressure. The effect of membrane tension is to shift V_h_, as indicated by the change in voltage at maximal capacitance as negative pressure increases, without appreciably altering frequency response (dotted lines are fits to a power function). On the right, C-V plots of complex capacitance magnitude at the four different pipette pressures are shown. Red bars indicate voltage at peak capacitance. The depolarizing shift in V_h_ is readily apparent as pressure increases. Peak magnitudes do not appreciably alter. ΔC_sa_ is tension and frequency independent. Mean +/− SEM (n = 8). From lowest to highest frequency fit values from Eq. m1 are (peak NLC, V_h_, z): Panel A, 179.15, 166.45, 146.43, 120.30, 88.90, 62.62, 41.51 fF; −49.4, −49.0, −49.0, −50.9, −49.0, −48.2, −51.2 mV; 0.70, 0.71, 0.71, 0.66, 0.70, 0.58, 0.53; Panel B, 182.80 175.62, 153.91, 124.40, 91.56, 60.38, 39.08 fF; −38.5, −40.0, −39.4, −40.7, −39.4, −36.4, 35.8 mV; 0.74, 0.72, 0.72, 0.67, 0.66, 0.60, 0.57. Panel C, 187.73, 175.16, 155.35, 124.08, 90.68, 57.97, 34.77 fF; −25.9, −25.7, −25.7, −26.2, −26.0, −20.6, −14.5 mV; 0.73, 0.73, 0.72, 0.67, 0.66,0.62, 0.65. Panel D, 185.03, 175.67, 156.00, 124.51, 91.22, 58.39, 36.32, fF; −17.2,−17.7, −18.0, −18.1, −8.8, −12.3, −8.0 mV; 0.67, 0.66, 0.66, 0.61, 0.60, 0.58, 0.55.

In Fig. 3, we explore the effects of membrane tension on the magnitude, $|{C}_{m}^{\ast }(\omega )|$, of complex NLC across frequency, as it is a robust measure of NLC, equivalent to that obtained with the phase tracking approach. Results at 0, −4, −8 and −10 mm Hg (i.e., 0, 0.53, 1.06 and 1.33 kPa) pipette pressure are shown in Fig. 3A–D, left panels. Membrane tension shifts V_h_, as indicated by the rearrangement of the capacitance magnitude traces as negative pressure alters. For example, in left panel A, the trace at −80 mV (green) is above the trace at 0 mV (red), whereas in left panel D, the positions are reversed. In the right panels, plots of C-V functions of complex capacitance magnitude at the four different pipette pressures are shown (mean +/− SEM, n = 8). The depolarizing shift in V_h_ is readily apparent as negative pressure increases (red bars). There is little change in peak capacitance, however. ΔC_sa_ is mainly unaffected by tension.

**Figure 4:**
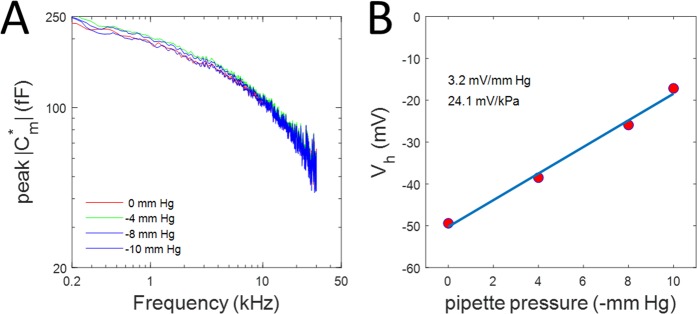
Effects of membrane tension on NLC frequency response and operating voltage. (**A)** Comparison of NLC frequency response at −40 mV at four pipette pressures. Roll-off is equivalent as indicated by overlap of traces. (**B)** V_h_ of NLC at the lowest frequency in Fig. 3 is plotted versus pipette pressure. A linear fit (blue line) indicates a sensitivity of 24.1 mV/kPa.

For comparison of frequency dependence as a function of membrane tension, Fig. 4A replots the peak magnitude traces (−40 mV) from Fig. 3 at the different pipette pressures. The frequency response of complex capacitance magnitude is unaffected by membrane tension, indicated by the overlap of traces. The sensitivity of patch V_h_ shift to tension was 24.1 mV/kPa based on our fits (Fig. 4B).

**Figure 5:**
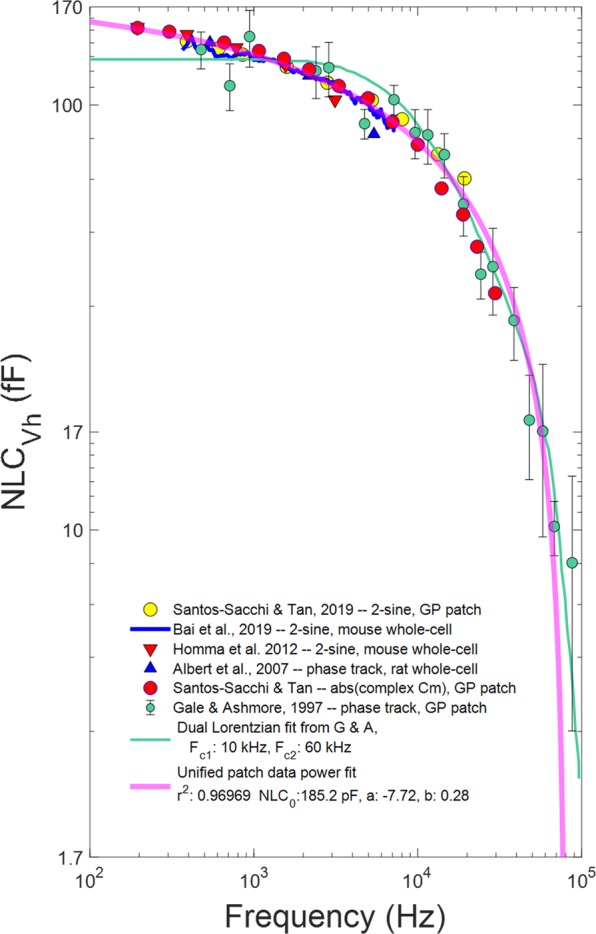
Replot of some whole-cell NLC data^27,45,46^, and NLC patch data from Santos-Sacchi and Tan^13^ with those of Gale and Ashmore^14^. Using a dual-sine capacitance estimation algorithm, NLC of OHC patches (yellow circles) overlie the data of Gale and Ashmore, collected with a traditional phase tracking approach using a lock-in amplifier (data: green circles; their fit green solid line). Whole cell data, both 2-sine and phase-tracking, show a similar frequency response within their interrogation bandwidth. Finally, NLC_Vh_ (red symbols) obtained from Eq. m1 fits of our complex capacitance data (obtained as in Fig. 2B) are also commensurate with previous observations, giving a unified power fit for the patch data (magenta line; see Discussion) with parameters NLC_0_: 185.2 fF, a: −7.22, b: 0.28.

We previously measured OHC NLC in macro-patches using a dual-sinusoidal methodology^13^. In Fig. 5, we replot selected NLC_Vh_ data points from that study (yellow circles) alongside NLC_Vh_ data (green circles) from Gale and Ashmore^14^. We also plot whole cell data on NLC_Vh_ (rat, mouse and guinea pig) from previous studies that used either the phase-tracking approach or 2-sine methodology. These scaled whole cell data interrogated a frequency bandwidth extending to 5 kHz, and closely follow the attenuation of patch NLC within that bandwidth. The Gale and Ashmore data (green circles, SEM, and fit from their Fig. 3B; in green) are also scaled (X 3.5) to overlie our data. Their measures were made with a lock-in amplifier using a 100 fF calibration at each frequency. Scaling to our data was necessary because our average patch size/capacitance was greater than theirs. As is evident, our previous data intersects their data points, indicating an acceptable correspondence. Finally, extraction of NLC_Vh_ from our new measures (red circles) of patch absolute complex capacitance (from Fig. 3, scaled X 1.05) corresponds well to our previous observations and theirs. We emphasize that the absolute magnitude of complex capacitance $({C}_{m}^{1}(\omega ))$ is equivalent to our phase tracking approach $({C}_{m}^{2}(\omega ))$ to estimate capacitance (Fig. 2), each corresponding to the estimates provided by the traditional phase tracking method^33^ employed by Gale and Ashmore^14^. In the discussion, despite their assertions, we detail how Gale and Ashmore’s data points above their 60 kHz amplifier cut-off are actually valid data points depicting NLC_Vh_. Capitalizing on the validity of their high frequency data, we combine all three patch data sets in the plot to arrive at a unified power function fit (r^2^ = 0.97) of NLC_Vh_ across frequency (solid magenta line), which shows precipitous roll-off of sensor charge movement relative to voltage drive. Fitted parameters are NLC_0_: 185.2 fF, a: −7.22, and b: 0.28. At 77 kHz, NLC_Vh_ is 40 dB down from NLC_0_. Furthermore, since each method takes into account the dielectric loss in prestin, we conclude that this dielectric loss does not significantly account for the low-pass nature of prestin charge movement. To further substantiate this claim, we make another estimate of membrane capacitance, ${C}_{m}^{3}(\omega )$, one that does not take into account dielectric loss, but is appropriate for a loss-less capacitor.

10 $${C}_{m}^{3}(\omega )=\,\frac{Im({Y}_{m}^{\ast }(\omega ))}{\omega }=\,\frac{{B}_{m}^{\ast }(\omega )}{\omega }$$

**Figure 6:**
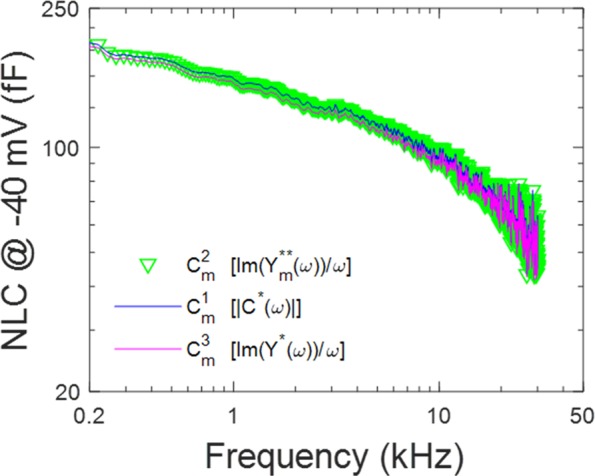
Three estimates of the frequency response of NLC at −40 mV holding potential. Estimates from raw admittance data (pink line) reasonably compare with the other estimates despite the fact that the real component is not taken into account.

Figure 6 shows the results for our three estimates of prestin capacitance [${C}_{m}^{1}(\omega ),\,{C}_{m}^{2}(\omega )\,and\,{C}_{m}^{3}(\omega )]$, all reasonably overlying each other, and exhibiting low-pass behavior. Thus, all results available to date show an unusually low-pass behavior of prestin voltage-sensor performance, with little influence of dielectric loss.

## Discussion

We previously evaluated OHC macro-patch NLC frequency dependence utilizing a dual-sine methodology that worked on extracted prestin displacement currents^13^. That approach did not allow separation of real and imaginary components of NLC. Here we provide an analysis of OHC NLC in membrane macro-patches utilizing single sine methods that have been used to characterize gating charge movements in other voltage-dependent proteins^30^, where real (capacitive) and imaginary (“resistive”, representing dielectric loss) components of sensor charge are separated. We explored these alternative approaches in order to determine whether new estimates of NLC frequency response incorporating the influence of dielectric loss would substantially alter our previous estimates, as was predicted through modelling^18^. This is not the case, however. Thus, the bulk of prestin charge movement appears to move in phase with voltage, and the small dielectric loss that we find does not significantly influence measures of OHC NLC frequency response.

It was previously noted that low frequency determined OHC NLC (C-V) measures are unable to identify whether prestin charge movement is multistate/diffusional in nature^8,34^. However, wide-band frequency interrogation of prestin NLC may provide clues. In this regard, the large bell-shaped (across frequency) resistive component of complex capacitance, which is associated with DPA^−^ voltage-driven translocation through bulk membrane lipid^35^, and which Sun *et al*.^18^ predicted for prestin modelled as a simple diffusion process of charge translocation is not found here. The resistive component we measure is relatively small and flat across frequency (Fig. 1A, lower panel). This is likely because prestin charge movement is not a diffusional process, but instead similar to gating charge movement in other membrane proteins. Thus, Fernandez *et al*.^36^ found that whereas chloroform, which alters membrane viscosity, can influence the speed of DPA^−^ charge translocation within the membrane, Na channel voltage-sensor charge translocation speed is not affected. Similarly, sensor charge in prestin is likely constrained to charged protein residues that move during protein conformational change^37^. Indeed, Sun *et al*.’s model included membrane diffusion of chloride ions as voltage sensors in prestin. However, chloride anions do not solely function as extrinsic voltage sensors for prestin, nor do they move through bulk lipid; instead they likely influence prestin kinetics^13,38,39^ (also see our comment #1 on Walter *et al*. eLife 8, 2019 - https://elifesciences.org/articles/46986)^40^.

Prestin’s voltage operating range, whose midpoint is at V_h_, is very sensitive to membrane tension^15^. Here, we measured the effect of membrane tension on prestin performance by changing patch pipette pressure to study its influence on NLC frequency response. The average sensitivity of patch V_h_ shift to tension was 24.1 mV/kPa, which corresponds well to our previous measures^17^ in whole cell mode of 21.6 mV/kPa. Adachi *et al*.^41^ found similar sensitivity in whole cell mode (27.5 mV/kPa). Gale and Ashmore^9^, in their patches, found a somewhat smaller sensitivity of 11.1 mV/kPa. In prestin transfected cells, values of near 4 mV/kPa were found^42,43^. One mechanism whereby shifts in V_h_ might occur would be due to changes in the ratio of transition rates between its conformational states. Naturally, if this were occurring then changes in NLC frequency response might arise. We found no change in NLC magnitude, similar to results of Gale and Ashmore^9^, or frequency response during alterations in membrane tension, and conclude that tension is not altering transition rates. Indeed, we previously found that membrane tension in whole cell mode did not significantly alter the phase or frequency response of electromotility^44^. This is in contrast to salicylate which likely alters transition rates since NLC frequency response is affected^13^.

Lastly, we revisited the seminal patch data of Gale and Ashmore^14^, along with those whole-cell NLC measures of others^27,45,46^, and recast our current and previous patch data^13^ with those prior results on OHC NLC. The small variability within our current and previous patch NLC data set allowed us to identify a power dependence of frequency, whereas Gale and Ashmore originally applied a Lorentzian fit. Their interpretation of NLC frequency response requires re-examination. They asserted that their capacitance measures made above their amplifier’s 60 kHz cut-off frequency were inaccurate, being unduly reduced by this cut-off. However, the amplifier’s transfer function effect on the phase tracking approach^24,31,33^ is inconsequential, since the lock-in phase is adjusted to maximize the imaginary (capacitive) component and minimize the real (resistive) component of the recorded signal. That is, the phase shift due to the amplifier was eliminated, and amplifier attenuation was corrected by their 100 pF calibration. Thus, their small-signal capacitance measurement was truly that of the patch membrane (and possibly non-voltage dependent stray capacitance), but when fitted by a Boltzmann derivative across voltage, provided valid, *not invalid*, estimates of voltage-dependent OHC NLC *above the amplifier cut-off*. Consequently, their fit to OHC NLC data at V_h_, which included the amplifier roll-off component, actually provided a dual-Lorentzian evaluation of OHC NLC, and their conclusion that only the lower frequency Lorentzian component of the fit fully characterized the behavior of OHC NLC (10 kHz cut-off) was misplaced. Furthermore, their interpretation suggested that OHC NLC roll-off is 6 dB/octave, when, in fact, roll-off is more than double that at high frequencies and is actually frequency dependent. Finally, the proposed inaccuracies in their high frequency estimates provided leeway to dismiss low-pass NLC indications of electromechanical behavior in favor of eM measures that were flat beyond 80 kHz^47^, the latter driving our concept of OHC eM’s wideband influence on cochlear amplification over the last couple of decades. In addition to all of our frequency evaluations of OHC NLC, past^13,20,23,48,49^ and present, recent eM measures, *in vitro* and *in vivo*, have challenged that concept^20,44,48,50^.

In summary, Fig. 5 shows that a power function fit to all patch data to-date reasonably describes the roll-off in NLC_Vh_. This unifying observation highlights an inability of voltage to sufficiently drive prestin electromechanical activity at very high frequencies (60–160 kHz), where CA (cochlear amplification) is known or expected to exist. The additional low pass influence of external loads on the whole cell eM frequency response and the frequency-dependent RC membrane time constant that we characterized recently^48^, also lessens the effectiveness of voltage-dependent eM in influencing organ of Corti motion at very high frequencies. This point is further emphasized by experiments showing that perilymphatic perfusion of 5 mM salicylate or its congener is devastating to CA^51,52^. Considering the 1.6 mM K_1/2_ of salicylate action, the effect of the perilymphatic treatment is not a full block of NLC, but rather about 75% (12 dB) reduction^53^. Thus, the reduction in NLC resulting from its power frequency dependence (12 dB down at 25 kHz, 20 dB down at 53 kHz, and 40 dB down at 77 kHz; Fig. 5) likely has biological impact *across the full auditory frequency spectrum of mammals*, and indicates a continuously diminishing influence of voltage alone to drive prestin electromechanical activity and CA via a cycle-by-cycle means. As a caveat, it may be possible that at any given frequency the measured NLC/eM amplitude is sufficient to drive CA, as we implied previously^20,54^, and that a reduction in CA that might occur with salicylate could be working on the existent magnitude at that particular frequency.

Two additional issues need to be addressed. They are temperature dependence, and species dependence of NLC frequency response. It is likely that temperature dependence of NLC frequency response is not so great (Q_10_ of 1.33), as Gale and Ashmore (1997) have directly attested to in membrane patches. We had previously argued that because V_h_ is highly temperature sensitive^55,56^, the kinetics of prestin should be, as well – we estimated a Q_10_ of 2 based on modelling of V_h_ shifts observed in IR temperature jump experiments^57^. But, since we now find that membrane tension does not influence prestin kinetics, in spite of shifts in V_h_, it is possible that temperature-induced shifts, likewise, work not through kinetics, but by some other means. Given that one-half magnitude NLC is at 7 kHz (from the power fit, Fig. 5), a Q_10_ of 1.33 would increase the cut-off to only about 10 kHz. Concerning species specificity, the data so far do not support such differences, since rat, mouse and guinea pig show similar frequency responses (see Fig. 5). Whether genetic variations in prestin that have been observed in mammals that capitalize on very high frequency detection^58,59^ could change this view remains possible, and we need to test these species. Currently, however, how CA is driven by OHCs in the basal high frequency turn of mammals that enjoy perception of sound above 60 kHz is an enigma.
